# Impact of Reduced Fluoroquinolone Use on *Clostridioides difficile* Infections Resulting From the Fluoroquinolone-Resistant Ribotype 027 Strain in a Veterans Affairs Medical Center

**DOI:** 10.20411/pai.v4i2.327

**Published:** 2019-10-01

**Authors:** Sarah N. Redmond, Sandra Y. Silva, Brigid M. Wilson, Jennifer L. Cadnum, Curtis J. Donskey

**Affiliations:** 1 Case Western Reserve University School of Medicine; Cleveland, Ohio; 2 Clinical and Translational Science Program; School of Medicine; Case Western Reserve University; Cleveland, Ohio; 3 Geriatric Research, Education, and Clinical Center; Louis Stokes Cleveland VA Medical Center; Cleveland, Ohio; 4 Research Service; Louis Stokes Cleveland VA Medical Center; Cleveland, Ohio

**Keywords:** fluoroquinolone, *Clostridioides difficile*, antimicrobial stewardship, environment, long-term care facility

## Abstract

**Background:**

Fluoroquinolone restriction has been proposed as a control measure for *Clostridioides difficile* infection (CDI) outbreaks associated with fluoroquinolone-resistant ribotype 027 strains. However, relatively few reports of fluoroquinolone restriction interventions have evaluated the impact on *C. difficile* strain types and fluoroquinolone resistance.

**Methods:**

In a hospital and affiliated long-term care facility (LTCF), antimicrobial stewardship and environmental cleaning interventions were implemented between 2009 and 2018, and *C. difficile* isolates during this period (~20 per year) were ribotyped and tested for fluoroquinolone resistance by moxifloxacin minimum inhibitory concentrations (MICs). Pearson's correlation coefficient was used to assess the association between use of fluoroquinolones and the percentage of CDI cases due to the 027 strain over time.

**Results:**

Between 2009 and 2018, prescribing of fluoroquinolones to inpatients decreased by 43%, coinciding with significant reductions in the healthcare-associated CDI rates in the hospital and LTCF and a decline in the percentage of *C. difficile* isolates that were ribotype 027 from 70% to 10%. Ninety-five percent of ribotype 027 and 6% of non-027 isolates were moxifloxacin resistant. Hospital fluoroquinolone use was strongly correlated with the incidence of hospital-associated CDI (r = 0.79, 95% confidence interval, 0.31-0.95), but LTCF fluoroquinolone use was not correlated with LTCF-associated CDI (r = 0.29, 95% confidence interval, -0.43-0.77). During the study period, there were statistically significant downward trends in the use of penicillins, intravenous vancomycin, carbapenems, and clindamycin.

**Conclusion:**

Our results provide support for fluoroquinolone restriction as a control measure for CDI outbreaks due to fluoroquinolone-resistant 027 strains, but also highlight the need for randomized trials as factors such as reduction in other antibiotic classes and improved cleaning may also impact CDI rates.

## INTRODUCTION

During the past 2 decades, the fluoroquinolone-resistant ribotype 027 strain of *Clostridioides difficile* has been associated with large outbreaks in North America and Northern Europe [1]. Evidence from mouse models and in patients suggests that exposure to fluoroquinolones results in selective pressure favoring colonization and infection with 027 strains [2–3]. Thus, restriction of fluoroquinolones has been proposed as a control measure for outbreaks associated with 027 strains [2–4]. In England, a national program that included reductions in the use of fluoroquinolones and cephalosporins in conjunction with improved infection control practices resulted in reductions in CDI that were driven by substantial decreases in fluoroquinolone-resistant genotypes, including 027 [4]. However, fluoroquinolone restriction has not always been similarly effective. For example, in a tertiary care hospital a significant reduction in fluoroquinolone prescribing did not reduce CDI or the proportion of cases due to 027 strains [5].

Given the importance of CDI as a public health problem, there is a need for additional evidence regarding the impact of fluoroquinolone prescribing on CDI rates and infections due to the 027 strain. At the Cleveland VA Medical Center, antimicrobial stewardship interventions during the past decade have included efforts to reduce overuse of fluoroquinolones, particularly in the affiliated long-term care facility (LTCF). Here, we tested the hypothesis that reductions in fluoroquinolone use in the hospital and LTCF would be associated with a reduction in the proportion of cases due to ribotype 027 strains.

## METHODS

### Protection of Human Research Participants

The institutional review board at the Cleveland VA Medical Center approved all study activities. Informed consent was waived.

### Setting and Interventions During the Study Period

The Cleveland VA Medical Center includes a 215-bed hospital and a 250-bed LTCF. Since January 2009, stool specimens testing positive for toxigenic *C. difficile* have been saved and stored at -80°C. Beginning in January 2009, an antimicrobial stewardship intervention in the LTCF reduced total antibiotic use with reductions in several classes of antibiotics, including fluoroquinolones [6]. In the hospital, antimicrobial stewardship interventions have included education focused on reducing the use of antibiotics considered high-risk for CDI, including fluoroquinolones, and reducing treatment of asymptomatic bacteriuria. Beginning in 2011, the infection control program implemented a cleaning and disinfection intervention that was effective in reducing environmental contamination with *C. difficile* [7].

To assess the impact of the stewardship interventions on fluoroquinolone use, we used pharmacy databases to assess systemic fluoroquinolone use measured as days-of-therapy per 1,000 patient-days in the hospital and LTCF. In the hospital, we also examined the use of cephalosporins (ceftriaxone, cefazolin, cefepime, cefoxitin), penicillins (ampicillin, ampicillin/sulbactam, piperacillin/tazobactam,), vancomycin (intravenous only), carbapenems (meropenem, imipenem-cilastatin, ertapenem), clindamycin, and macrolides (azithromycin, clarithromycin). CDI rates were measured as healthcare facility-associated (HCFA) CDI cases per 10,000 patient-days for the hospital and LTCF.

### Molecular Typing of *C. difficile* Isolates

To estimate the percentage of HCFA CDI cases due to the 027 strain, we cultured the first 5 individual patient stool specimens from each quarter of the year (a total of 20 per year) from 2009 through 2018 for toxigenic *C. difficile* [5, 7]. PCR ribotyping using capillary gel electrophoresis and fluorescent ribotyping was performed as previously described [5]. All isolates were tested for moxifloxacin susceptibility by broth dilution minimum inhibitory concentrations (MIC). Strains were classified as susceptible (MIC ≤ 2ug/uL), intermediate (MIC 4ug/uL), resistant (MIC >8ug/ uL), and highly resistant (MIC > 32ug/uL) [2].

### Statistical Analysis

We calculated the percentage of CDI isolates each year that were due to the 027 strain. We used Pearson's correlation coefficient to assess the association between fluoroquinolone use and the percentage of CDI cases due to the 027 strain over time. The non-parametric Mann-Kendall test was used to detect monotone trends in the CDI rate and use of antibiotics. All analyses were performed using R version 3.5.1 statistical software (The R Foundation for Statistical Computing, Vienna, Austria).

## RESULTS

**Figure 1.**
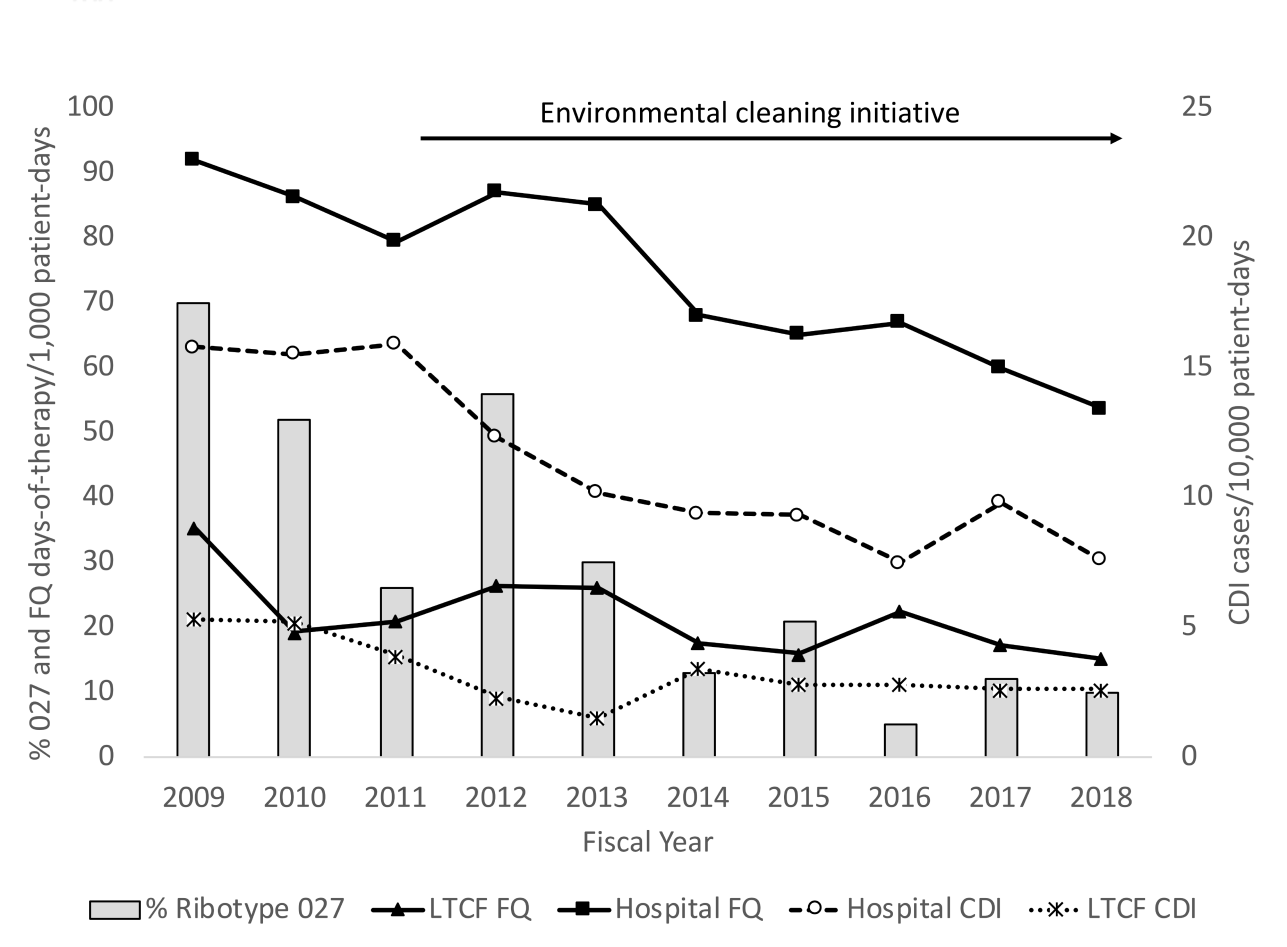
Changes in the incidence of hospital- and long-term care facility (LTCF)-associated *Clostridioides difficile* infection (CDI) and in the percentage of CDI infections due to ribotype 027 strains during a period of decreasing fluoroquinolone use. The environmental cleaning initiative resulted in reduced environmental contamination with *C. difficile*. Stewardship interventions also resulted in reductions in hospital use of penicillins, carbapenems, and intravenous vancomycin during the study period. Note. FQ, fluoroquinolone.

Figure 1 shows the rates of hospital- and LTCF-associated CDI cases and fluoroquinolone use as well as the percentage of *C. difficile* isolates that were ribotype 027. The HCFA CDI rate decreased in the hospital and LTCF by 52% and 51%, respectively. Prescribing of fluoroquinolones to inpatients decreased by 43% overall from 2009 to 2018, with 57% and 42% reductions in the hospital and LTCF, respectively. The reduction in fluoroquinolone use coincided with a decline in percentage of *C. difficile* isolates that were ribotype 027 from 70% to 10%. The decrease in CDI and in the percentage of cases due to ribotype 027 also coincided with the environmental cleaning initiative [7]. Mann-Kendall trend analysis demonstrated that the decreases in the CDI rate and in fluoroquinolone use were statistically significant in both the hospital and LTCF (*P* ≤ 0.02). Hospital fluoroquinolone use was strongly correlated with the incidence of hospital-associated CDI (Pearson r = 0.79, 95% confidence interval 0.31-0.95; *P* < 0.01), but LTCF fluoroquinolone use was not correlated with LTCF-associated CDI (Pearson r = 0.29, 95% confidence interval -0.43-0.77; *P* = 0.44).

During the study period, hospital cephalosporin use increased by 45% (53 to 76 days-of-therapy per 1,000 patient-days), but the trend in use was not statistically significant (*P* = 0.86). There were statistically significant downward trends in the use of penicillins (17% decrease from 123 to 102 days-of-therapy per 1,000 patient-days, *P* < 0.01), intravenous vancomycin (36% decrease from 103 to 66 days-of-therapy per 1,000 patient-days, *P* < 0.01), carbapenems (44% decrease from 19 to 11 days-of-therapy per 1,000 patient-days; *P* < 0.01), and clindamycin (25% decrease from 4 to 3 days-of-therapy per 1,000 patient-days; *P* = 0.02). Macrolide use decreased by 18% from 22 to 18 days-of-therapy per 1,000 patient-days, but the trend in use was not statistically significant (*P* = 0.07).

**Figure 2.**
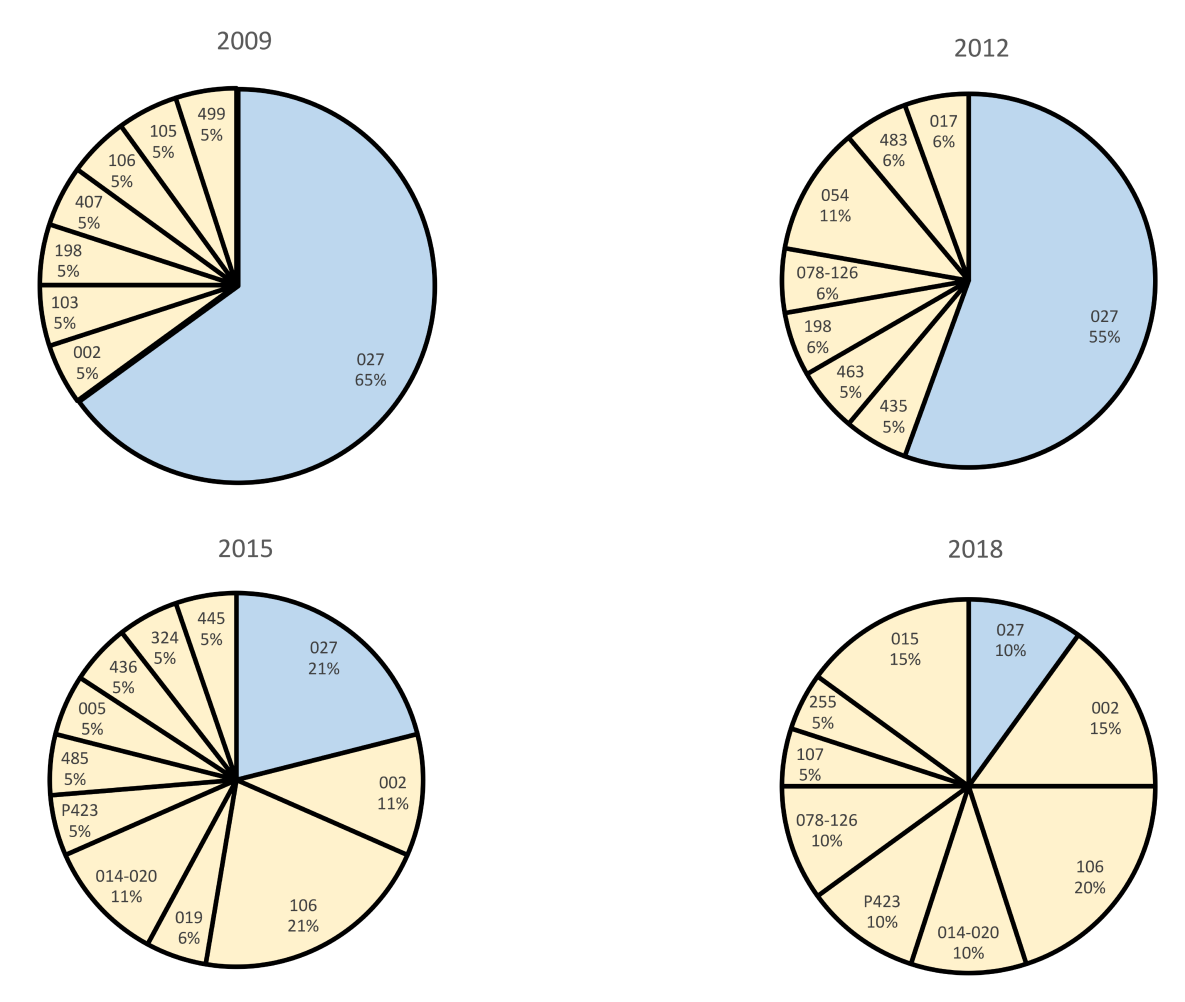
Distribution of polymerase chain reaction ribotypes of *Clostridioides difficile* isolates recovered from stool specimens testing positive for *C. difficile* during 4 representative years in a 10-year period. There were a total of 203 isolates (20 to 23 per year) subjected to ribotyping.

Figure 2 shows the distribution of *C. difficile* ribotypes for 4 representative 1-year periods during the study. The decline in ribotype 027 was not associated with emergence of another predominant strain, rather a variety of strain types were detected. Strains that emerged included 014-020 and 078-126. Of 61 ribotype 027 isolates, 58 (95%) were moxifloxacin-resistant, including 29 (48%) that were highly resistant. Only 8 of 142 (6%) non-027 strains were fluoroquinolone resistant and none were highly resistant. The 8 fluoroquinolone-resistant non-027 isolates were from 6 different ribotypes including F053-163 (N = 2 isolates), F001 (N = 2 isolates), F017, F014-020, F002, and F198.

## DISCUSSION

We found that antimicrobial stewardship interventions in our facility during the past decade have resulted in significant reductions in the use of fluoroquinolones, penicillins, carbapenems, and intravenous vancomycin, but not cephalosporins. An intervention to improve cleaning and disinfection was also effective in reducing environmental contamination with *C. difficile* [7]. We demonstrate that these interventions have been associated with reduced CDI incidence and a dramatic decrease in infections due to ribotype 027 strains. A majority of 027 strains tested were resistant to moxifloxacin, whereas only 6% of non-027 strains were moxifloxacin resistant.

Our findings provide support for fluoroquinolone restriction as a control measure for CDI, particularly in settings where epidemic 027 or other fluoroquinolone-resistant strains are common causes of infection. It is microbiologically plausible that the reduction in the 027 strain is attributable to reduced fluoroquinolone selective pressure. However, we cannot exclude the possibility that other factors may have contributed. If the 027 strain is more likely to spread or cause symptomatic disease, infection control interventions might have a disproportionate effect on these strains. In addition, epidemic *C. difficile* strains may decline in the absence of antibiotic restriction [3]. Recent data suggest that the prevalence of CDI due to the 027 strain may be decreasing throughout the VA healthcare system [8], and although fluoroquinolone use has decreased, this was not significant [9]. Previous quasi-experimental studies that have reported reductions in CDI and in the proportion of 027 strains in association with fluoroquinolone restriction have similar limitations as reductions in other antibiotic classes and improvements in cleaning occurred in conjunction with reductions in fluoroquinolones [3–4].

Although it is likely that antimicrobial stewardship interventions contributed substantially to the reduction in fluoroquinolone use in our facility, other factors could have played a role in reducing the use of these agents. Clinicians may have reduced prescription of fluoroquinolones in response to concerns about the risks associated with fluoroquinolones and awareness of increasing levels of resistance among Gram-negative pathogens in the facility. The US Food and Drug Administration (FDA) issued warnings regarding serious side effects associated with fluoroquinolones in 2008 and 2013 and enhanced warnings were published in 2016 [10]. The 2016 FDA communication recommended that fluoroquinolones should not be used for patients with acute bacterial sinusitis, acute bacterial exacerbation of chronic bronchitis, and uncomplicated urinary tract infections unless no alternative treatment options were available [10]. We have previously reported increasing rates of fluoroquinolone resistance among *Escherichia coli* urinary tract isolates from our facility [11–13]. Urologists and primary care providers were alerted to the problem of increasing fluoroquinolone resistance in 2010 due to an outbreak of infections with fluoroquinolone-resistant *E. coli* after transrectal ultrasound-guided prostate biopsy [11–12]. Others have reported reductions in the use of fluoroquinolones during the period of our study [9, 14–15].

In contrast to our findings, Hecker *et al* [5] reported that a similar reduction in fluoroquinolone prescribing in a tertiary care hospital was not associated with a reduction in the incidence of healthcare-associated CDI or in the proportion of cases due to 027 strains [5]. One potential explanation for the differing findings might be the difference in the setting of the studies or the patient populations. The study of Hecker *et al* [5] was conducted in a tertiary care level 1 trauma center receiving patients from many outside referral centers that might not have had similar reductions in fluoroquinolone use. The Cleveland VA Medical Center provides care for a more homogenous population and stewardship interventions and changes in practice patterns might be more likely to be implemented for the entire patient population. In addition, the baseline healthcare-associated CDI rate was substantially lower in the tertiary care hospital than in the Cleveland VA Medical Center [5]. It is plausible that reductions in antibiotic use might have a greater impact in settings with higher initial CDI rates.

Our study has some limitations. The study was conducted in a single healthcare facility with predominantly male and elderly patients. As noted previously, multiple potential confounding factors could have impacted CDI rates and the prevalence of 027 strains. We did not examine the appropriateness of fluoroquinolone therapy. However, substantial reductions in fluoroquinolone use may be feasible because these agents are often prescribed unnecessarily [5, 16].

## CONCLUSION

We found that reductions in fluoroquinolone use were associated with reductions in the incidence of CDI and in infections due to ribotype 027 strains in a Veterans Affairs Medical Center. However, confounding factors such as reductions in other classes of antibiotics and improvements in environmental cleaning occurred during the study period. Thus, randomized trials are needed to clarify the effectiveness of fluoroquinolone restriction as a control measure for CDI.
